# Downregulated GABA and BDNF-TrkB Pathway in Chronic Cyclothiazide Seizure Model

**DOI:** 10.1155/2014/310146

**Published:** 2014-03-13

**Authors:** Shuzhen Kong, Zhihua Cheng, Jianhui Liu, Yun Wang

**Affiliations:** ^1^Institutes of Brain Science and State Key Laboratory of Medical Neurobiology, Fudan University, Shanghai 200032, China; ^2^Chongqing Key Laboratory of Catalysis and Functional Organic Molecules and Chongqing Key Laboratory of Nature Medicine Research, Chongqing Technology and Business University, Chongqing 400067, China; ^3^Department of Neurosurgery, Shanghai Ninth People's Hospital, School of Medicine, Shanghai Jiao Tong University, Shanghai 200011, China

## Abstract

Cyclothiazide (CTZ) has been reported to simultaneously enhance glutamate receptor excitation and inhibit GABAA receptor inhibition, and in turn it evokes epileptiform activities in hippocampal neurons. It has also been shown to acutely induce epileptic seizure behavior in freely moving rats. However, whether CTZ induced seizure rats could develop to have recurrent seizure still remains unknown. In the current study, we demonstrated that 46% of the CTZ induced seizure rats developed to have recurrent seizure behavior as well as epileptic EEG with a starting latency between 2 weeks and several months. In those chronic seizure rats 6 months after the seizure induction by the CTZ, our immunohistochemistry results showed that both GAD and GAT-1 were significantly decreased across CA1, CA3, and dentate gyrus area of the hippocampus studied. In addition, both BDNF and its receptor TrkB were also decreased in hippocampus of the chronic CTZ seizure rats. Our results indicate that CTZ induced seizure is capable of developing to have recurrent seizure, and the decreased GABA synthesis and transport as well as the impaired BDNF-TrkB signaling pathway may contribute to the development of the recurrent seizure. Thus, CTZ seizure rats may provide a novel animal model for epilepsy study and anticonvulsant drug testing in the future.

## 1. Introduction

Gamma-aminobutyric acid (GABA) is the primary inhibitory neurotransmitter and exists widely in central nervous system of mammal animals. GABA receptors mediate inhibitory neurotransmission to prevent neurons from being overexcited in adult brain [1]. GABA is synthesized from glutamate by glutamate decarboxylase (GAD), which is a rate-limiting enzyme in GABA synthesis. Then GABA is released from neurons to synaptic cleft and exerts inhibitory effect. The major cortical GABA transporters (GAT), which uptake the GABA to neurons or glia from synaptic cleft, primarily localized to the presynaptic terminals and to glial processes adjacent to the synaptic cleft. Meanwhile, GAT also has been reported to participate in GABA releasing to adjust GABA concentration in synaptic cleft [2].

Brain-derived neurotrophic factor (BDNF) is a member of the neurotrophin family and mainly exists in hippocampus, amygdale, cortex, and cerebellum. It is synthesized by both neurons and glia and involved in survival, differentiation, and regeneration of neurons by binding its high affinity receptor, TrkB [3, 4]. BDNF is found in hippocampus which is highly sensitive for epileptiform insults [5–7]. However, there are still many controversial viewpoints about the role of BDNF in epileptogenesis.

Cyclothiazide (CTZ) not only blocks AMPA receptor desensitization, but also inhibits GABAA receptors [8], which leads to induction of epileptiform activities in hippocampal neurons in both in vitro and in vivo preparations [7, 9–11]. CTZ has also been demonstrated to acutely induce seizure behavior and epileptic EEG in freely moving rats [12], which involves extrasynaptic GABAA receptor participation [13]. Thus, CTZ is thought to be a useful convulsant for seizure animal model generation. However, whether CTZ could induce chronic seizure and the property of CTZ induced chronic seizure have still not been fully investigated. Here, we firstly reported the observation of a high proportion of the acutely CTZ induced seizure rats having recurrent seizure behavior during the period of up to 6 months after CTZ injection. We also demonstrated that the recurrent seizure induced by CTZ was associated with the typical epileptic EEG. The immunohistochemistry results further showed that the occurrence of the recurrent seizure was likely mediated by the impaired GABA inhibitory transmitter system and BDNF-TrkB pathway.

## 2. Methods

### 2.1. Animals

Adult male Sprague-Dawley rats (250–280 g; supplied by the Research Institute of Surgery, Chinese Third Military Medical University, Chongqing, China) were used for seizure induction and behavioral observation. All animal experiments were approved by the local committee of Laboratory Animals Usage of Chongqing Business and Technology University and carried out in accordance with Chinese National Science Foundation animal research regulation.

### 2.2. Behavioral Test and EEG Recording

Acute seizure induced by i.c.v. injection of CTZ was described in detail preciously [12]. 13 kindled seizure rats induced by CTZ (0.25 *μ*mol in 5 *μ*L, i.c.v.) injection, with behavioral seizure of grade IV and V, and 8 control rats treated with DMSO (5 *μ*L, i.c.v.) were recorded for both behavior and EEG for 2 hours every other day until the 6th month. In two cases, the behavioral observation was carried out for continuous 6 months; both rats showed multiple recurrent seizure episodes. Behavioral seizures were scored using 5-graded Racine score system as previously defined [14]: briefly, Racine score I, facial clonus; score II, head nodding; score III, unilateral forelimb clonus; score IV, rearing with bilateral forelimb clonus; score V, rearing and falling (loss of postural control). The recurrent seizure assessment was based on the latency for spontaneous seizure behavior, the seizure score, and the seizure number. The seizure score in an animal was calculated based on the maximal behavioral seizure grade observed [15, 16].

For EEG recordings, electrophysiological signals were amplified (×1000) and filtered (0–50 Hz) using NeuroLog system (Digitimer Ltd, Hertfordshire, UK), digitized with CED Micro 1401 (Cambridge Electronic Design, Cambridge, UK), and recorded in a personal computer using Spike 2 software (version 6.0, Cambridge Electronic Design, Cambridge, UK). The presence of high-amplitude EEG seizure activity was used as a seizure marker. In some cases, the EEG recordings were unable to continue for the whole 6-month recording period due to their loss of the implanted skull electrodes.

### 2.3. Tissue Preparation

At the end of the 6-month recording, all rats, except those which died earlier, were perfused through the ascending aorta with 0.9% NaCl rapidly followed by 200 mL 4% paraformaldehyde in phosphates buffer. The brains were postfixed in the same fixative overnight at 4°C and were embedded in paraffin after dehydration with alcohol of different concentration. 6 mm thick sections were cut in a microtome and mounted on poly-L-lysine coated slides. The CTZ rats exhibited grade IV or V recurrent seizure behavior and the DMSO rats were selected for immunohistochemistry staining.

### 2.4. Nissl Staining

Four to six slides for every rat were deparaffinated with xylene and alcohol of different concentration, rinsed in tap water and then in distilled water, stained in 0.1% thionine solution for 10 minutes, rinsed quickly in distilled water, differentiated in 95% HCl-alcohol for 2–10 minutes, dehydrated in alcohol of different concentration for 5 minutes every time, cleared in xylene for 5 minutes, and mounted with neutral balsam solution.

### 2.5. Immunohistochemistry

SP immunohistochemistry assay for different receptor subunits was performed. Four to six slides from each rat were deparaffinated with xylene and alcohol of different concentration and incubated with 3% H_2_O_2_. Antigens of sections were retrieved with 0.125% trypsin solution. Sections were rinsed in Tris-HCl-buffered saline and preincubated with normal goat serum (SP-9001, ZSGB-BIO, China), followed by incubation with the primary antibodies, anti-GAD65 + GAD67 (Abcam), anti-GAT1 (Abcam), anti-BDNF (Abcam), and anti-TrkB (Abcam), at 4°C overnight. Subsequently, they were incubated with horseradish peroxidase-coupled goat anti-rabbit secondary antibodies (SP-9001, ZSGB-BIO, China) for 60 min at 37°C temperature. Finally, the sections were reacted with 0.4 mM 3,30-diaminobenzidine (ZSGB-BIO, China) and 0.01% H_2_O_2_ for 10–15 min. After each incubation step, except the preincubation, three 5 min washes with Tris-HCl-buffered saline were performed.

### 2.6. Image Acquisition and Analysis

Bright field images were acquired digitally on a Nikon 80i microscope with an NIS-elements 3.0 software (Nikon, Japan). Quantitative analysis of number of neurons in Nissl staining was performed manually and the numbers of GAD, GAT1, BDNF, and TrkB positive cells were quantitatively analyzed with NIS-elements 3.0 software.

### 2.7. Data Analysis

All data were expressed as mean ± SEM. Comparison between the CTZ group and the DMSO group was executed with independent-sample *t*-test. Results were considered significant at *P* < 0.05.

## 3. Results

### 3.1. Recurrent Seizure Behavior and Epileptic EEG of Chronic CTZ Seizure Model Rats

**Figure 1:**
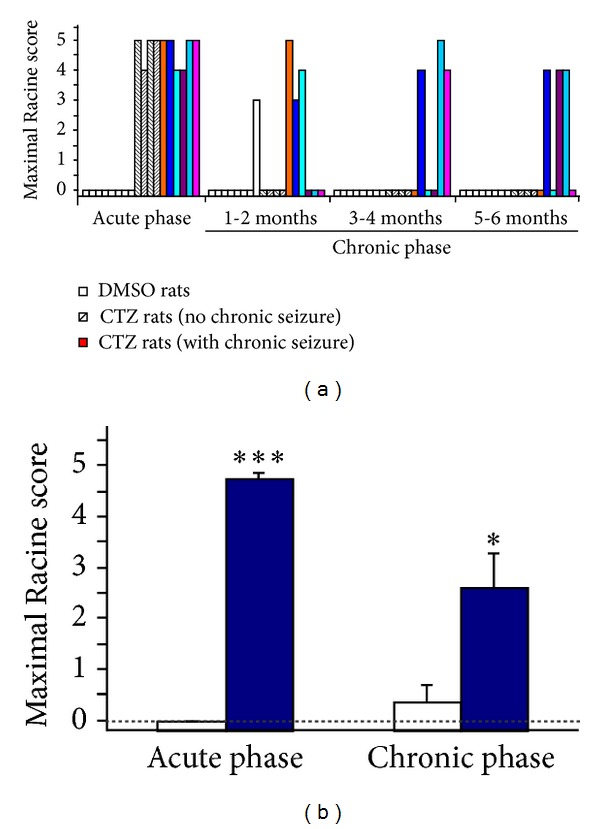
Recurrent seizure behavior occurrence in the chronic phase after CTZ induced seizure rats. (a) Bar chart showing the seizure behavior occurrence within 6-month period after CTZ induced acute seizure in individual rats studied. DMSO control rats (*n* = 8) were represented with white bar and CTZ rats (*n* = 10) were represented with either black strip bars (*n* = 4) or color bars (*n* = 6) for rats without or with recurrent seizure observed, respectively. (b) Bar chart showing the group maximal seizure scores obtained from the DMSO control rats (*n* = 8) and CTZ rats (*n* = 10) recorded for 6 months after CTZ induction of the seizure (*P < 0.05, ***P < 0.001, in comparison with the DMSO control).

Previously we have reported that intracerebroventricular administration of CTZ acutely induced robust epileptic seizure [12]. Here we further studied the seizure behavior in 13 CTZ induced seizure rats for their chronic seizure activities for a period up to 6 months after CTZ administration in a noncontinuous monitoring experimental paradigm (see Section 2). In these 13 rats, they all had Racine score 4 and score 5 seizure behavior during the acute seizure induction phase (Figure 1). Among them, 6 (46.2%) had been observed to have recurrent seizure behavior with the shortest latency of 15 days (mean latency of 76.3 ± 24.8 days) after CTZ injection; 4 of them were without recurrent seizure being observed (Figure 1(a)); and 4 other rats were dead at days 6, 9, 15, and 99 after the CTZ injection (including 1 that had generalized seizure one day before its death at day 15). Recurrent seizure behavior showed as blinking, salivating, facial or automatisms, forelimb clonus, and even rearing and falling, similar to those behaviors showed during the acute phase [12]. Among those 6 rats observed to have recurrent seizure behavior, we found 2 rats having multiple seizure episodes (4 and 3, resp.) within 6-month recording period (Figure 1(a)), and the mean maximal Racine score for those 6 rats having recurrent seizures was 4.33 ± 0.16 (*n* = 6, Figure 1(b)), similar to the acutely induced seizure behavior (4.75 ± 0.16, *n* = 6) in these 6 rats. In contrast, 8 DMSO (i.c.v.) administered rats during acute seizure induction phase were not observed to have any seizure behavior, and only 1 rat had a stage 3 behavior at day 14 after injection, but not in the other 7 rats for recording up to 6 months (Table 1). As a group (*n* = 10 including those having and not having recurrent seizure but without 3 dead rats), the mean maximal recurrent seizure score was 2.60 ± 0.72 in those CTZ rats, which significantly differed to the DMSO control group (0.38 ± 0.38, *n* = 8; *P* < 0.05) (Figure 1(b)).

**Figure 2:**
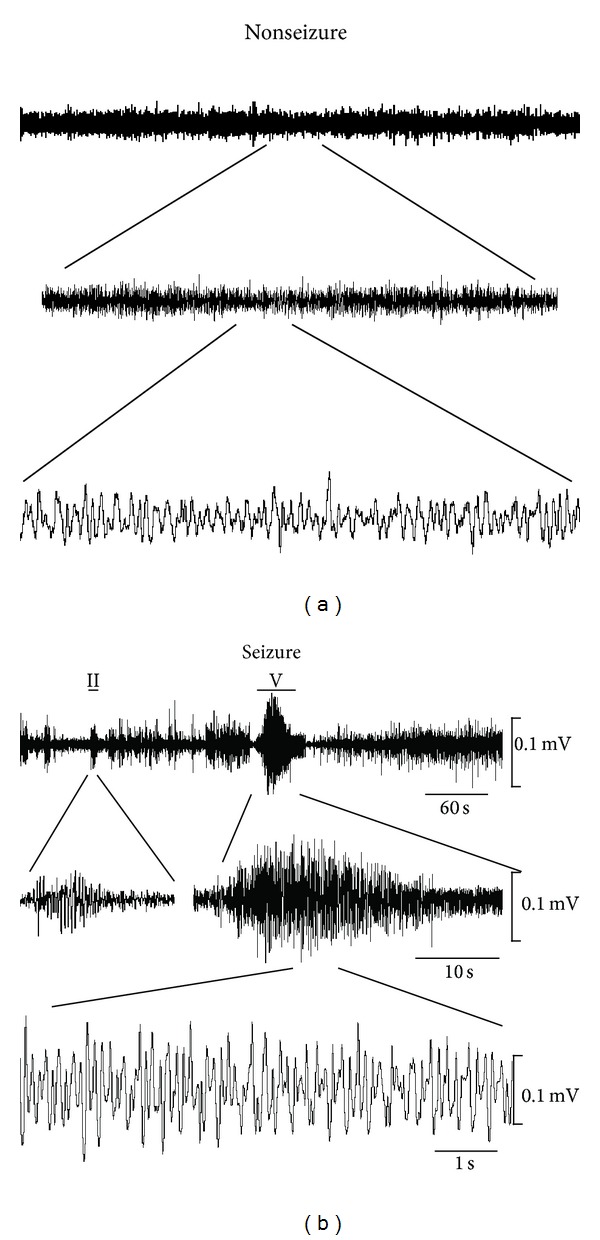
Epileptic EEG recorded in the chronic phase of a CTZ induced seizure rat. Raw EEG recording traces showing (a) normal EEG waveform during nonseizure period and (b) high amplitude high frequency interictal and ictal like epileptic EEG associated with the seizure behavior (marked with bars above the EEG traces) from CTZ induced seizure rats at the time of 6 months after initial seizure induction by CTZ.

In addition to the seizure behavior observed in the chronic phase, we also successfully recorded the EEG response in 3 out of 6 of the CTZ induced seizure model rats at the certain period within the 6-month observation period. As shown in Figure 2, the epileptiform EEG activities were detected on day 150 after initial seizure induction in one rat. The ictal like high amplitude high frequency bursting activities were usually associated with the typical seizure behaviors with the Racine score III or above (Figure 2(b)). In addition, interictal spikes or sharp waves also appeared before or after the ictal like bursting EEG activities (Figure 2(b)). However, neither interictal spikes nor ictal like bursting EEG activities were observed during the period while the seizure behavior was absent (Figure 2(a)).

Above all, CTZ seizure models exhibited recurrent seizure behaviors associated with epileptic EEG changes in chronic phase 1–6 months after acute seizure induction.

### 3.2. CTZ Seizure Did Not Affect the Hippocampal Neuronal Fate for up to 6-Month Chronic Recurrent Phase in Rats

**Figure 3:**
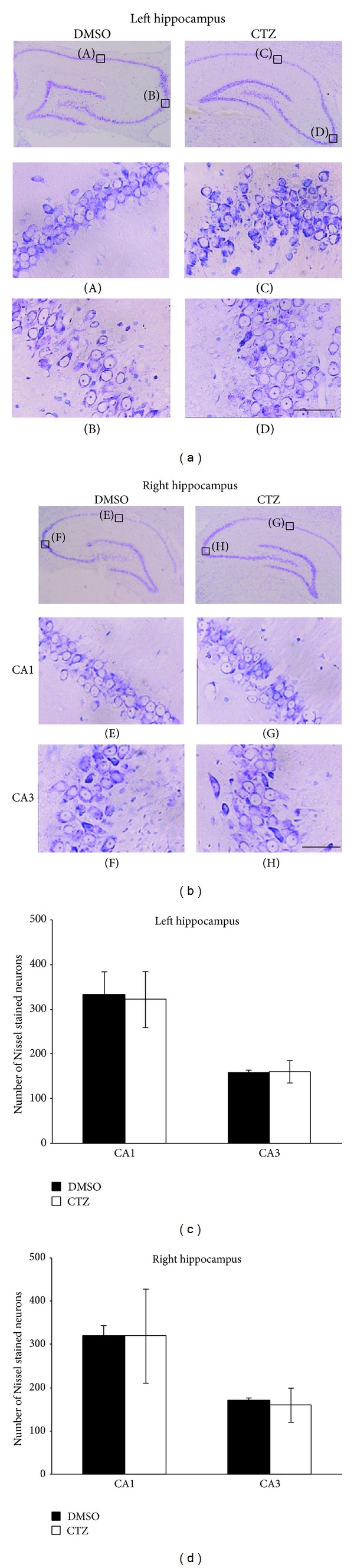
Nissl staining of the hippocampal neurons in chronic CTZ seizure rats 6 months after seizure induction. (a-b) Pictures showing the Nissl staining of the left (a) and right (b) hippocampus of either DMSO or CTZ treated rats. The expanded pictures (A–H) showing the detail of the Nissl stained hippocampal CA1 and CA3 neurons. (c-d) Group data of the counted Nissl stained hippocampal neurons in left (c) and right (d) hippocampus of either DMSO or CTZ chronic seizure rats. Scale bar = 50 *μ*m.

Nissl staining was used to investigate the morphological alteration and neuron loss [17]. In our current study, Nissl staining was performed in the hippocampal slices taken from 3 CTZ rats with chronic seizure score of grade VI or V and also 3 DMSO rats without chronic seizures 6 months after either DMSO or CTZ injection. It showed that undamaged Nissl positive neurons in both CA1 and CA3 of either left or right hippocampus had similar number count (Figure 3). There were also some damaged neurons, but with the minority in count, in all the areas including CA1, CA3, and DG, which showed incomplete forms, cell swelling or shrinkage, vague outlines, and confused boundaries with Nissl staining. The Nissl positive neurons in left CA1 and CA3 in DMSO control rats were 333 ± 54 and 158 ± 8 (per section of the area counted) and in CTZ treated rats were 322 ± 64 and 161 ± 27, respectively. The corresponding number counts for the right CA1 and CA3 were 320 ± 27 and 171 ± 9 for DMSO control and 321 ± 111 and 161 ± 42 for CTZ rats, respectively.

This result demonstrated that CTZ itself did not have profound effect on the cell death seen in the other major convulsant induced seizure animal models, which was consistent with the previous notion suggested according to the in vitro work [9].

### 3.3. Decreased GAD Staining in Hippocampus in Chronic CTZ Seizure Model Rats

**Figure 4:**
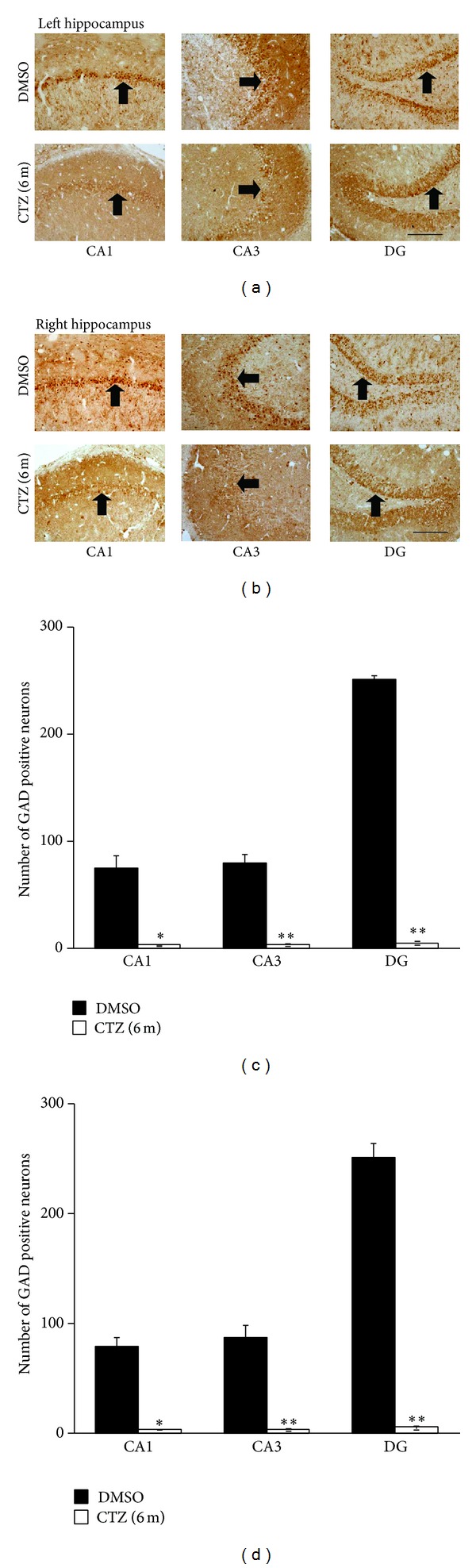
Decreased GAD staining in hippocampus of recurrent seizure rats 6 months after seizure induction by CTZ. (a-b) Pictures showing GAD positive cells (arrow indicated) in left (a) and right (b) hippocampus from rats 6 months after either DMSO (top) or CTZ (bottom) treatment. (c-d) Group data showing significant decreases of the GAD staining cells in CA1, CA3, and DG area of the hippocampus from rats 6 months after either DMSO (*n* = 3) or CTZ (*n* = 3) treatment. *P < 0.05, **P < 0.01 compared to the DMSO control group. Scale bar in (a) and (b) = 200 *μ*m.

GABA deficiency is one of the reasons for imbalance between excitation and inhibition associated with the seizure induction. Previous studies have indicated that CTZ induced seizure is likely generated from hippocampus [7, 9, 10]. In this aspect, we investigated whether GABAergic neurons in the hippocampus were also affected during epileptogenesis in its chronic phase, by immunostaining of GAD, a rate-limiting enzyme in GABA synthesis, and GAT-1, a cell membrane localized GABA transporter [18–20]. GAD positive immunohistochemistry staining brown particles were observed to exist in cytoplasm of the cells. GAD positive neurons distributed densely adjacent to pyramidal cell layer of CA1 and CA3 subregion and adjacent to granule cell layer of DG subregion of hippocampus in DMSO treated rats, and there are also some diffused GAD positive neurons in molecular layer and oriens layer of the hippocampus (Figures 4(a) and 4(b)). In comparison with the DMSO control rats, GAD positive particle containing neurons were also observed to localize in CA1, CA3, and DG area of CTZ induced seizure rats, but with significantly less amount. The number of GAD positive neurons in CA1, CA3, and DG was significantly decreased from 75.2 ± 13.0 in CA1, 79.7 ± 9.7 in CA3, and 251.5 ± 4.3 in DG, respectively, in the time matched DMSO control rats (*n* = 3) (mean ± SEM in counted area per section, see above Method) to 3.0 ± 0.5 in CA1, 3.6 ± 0.9 in CA3, and 5.3 ± 1.8 in DG in left hippocampus of 3 CTZ rats with recurrent seizure 6 months after CTZ injection (*P* < 0.05, *P* < 0.01) (Figures 4(a) and 4(c)). Further, the reduction of the GAD positive particles in either left or right brain had no obvious preference (Figures 4(c) and 4(d)), despite the fact that CTZ was injected in the left cerebral ventricle during acute seizure induction phase [12].

GAD represents the ability of GABA synthesis and GAD positive neurons are regarded as GABAergic neurons [21]. Thus, the above results suggest that GABA in hippocampus became reduced in chronic phase of the CTZ induced epileptic seizure, which may lead to a deficiency of GABA inhibition. The decline in DG of the GAD positive neurons in CTZ seizure models was most obvious, which suggest that the GABAergic neurons in this region was likely damaged most seriously.

### 3.4. Decreased GAT in Neurons of Hippocampus in CTZ Seizure Rats in Chronic Recurrent Phase

**Figure 5:**
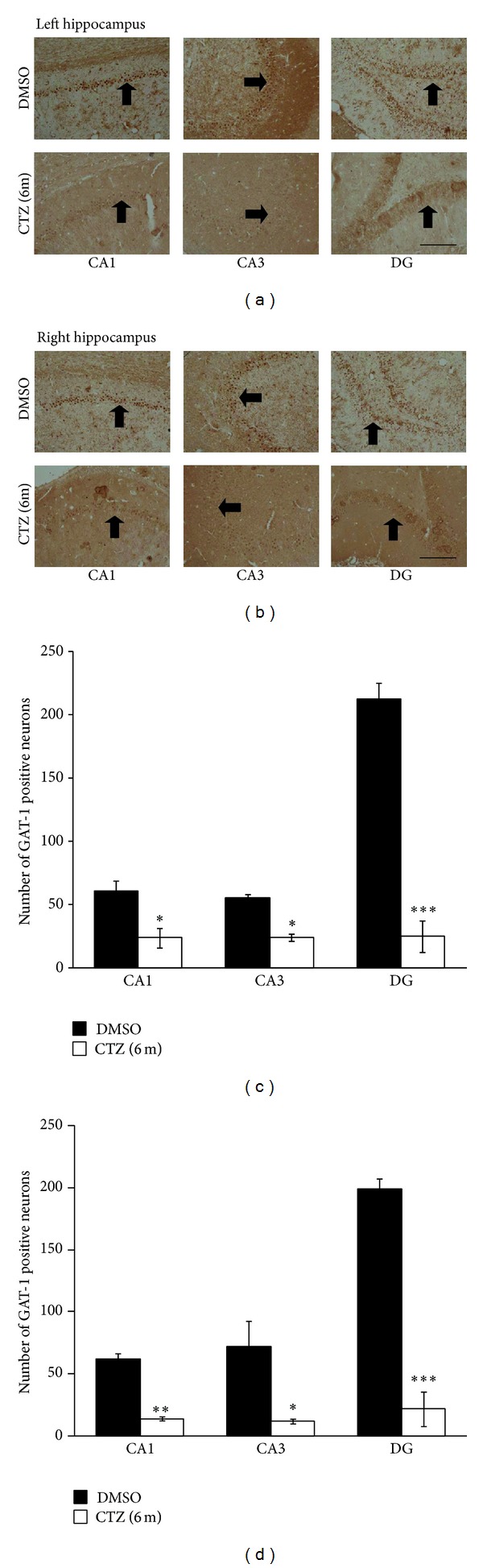
Decreased GAT-1 staining in hippocampus of recurrent seizure rats 6 months after seizure induction by CTZ. (a-b) Pictures showing GAT-1 positive cells (arrow indicated) in left (a) and right (b) hippocampus from rats 6 months after either DMSO (top) or CTZ (bottom) treatment. (c-d) Group data showing significant decreases of the GAT-1 staining cells in CA1, CA3, and DG area of the hippocampus from rats 6 months after either DMSO (*n* = 3) or CTZ (*n* = 3) treatment. *P < 0.05, **P < 0.01, and ***P < 0.001 compared to the DMSO control group. Scale bar in (a) and (b): 200 *μ*m.

In addition, GAT-1 stained positive particles were also seen in the cytoplasm of the cells located adjacent to the pyramidal cell layer of CA1 and CA3 subregion and the granule cell layer of DG subregion of the hippocampus in DMSO control rats. Similar to the GAD staining, GAT-1 positive neurons were also significantly reduced in CA1, CA3, and DG regions in the CTZ seizure rats (Figures 5(a) and 5(b)). The number of the GAT-1 positive neurons were counted as 20.7 ± 8.6 in CA1, 24.3 ± 3.4 in CA3, and 24.7 ± 13.3 in DG (mean ± SEM in counted area per section, see above Method) in left hippocampus of 3 CTZ rats with recurrent seizure 6 months after CTZ injection, which was significantly less than those of time matched DMSO control rats (60.7 ± 3.0 in CA1, 55.7 ± 9.1 in CA3, and 212.3 ± 11.3 in DG, resp.; *n* = 3) (*P* < 0.05, *P* < 0.001) (Figures 5(a) and 5(c)). Similar to the result of GAD staining, the proportion of the decreased GAT-1 positive neuron had no obvious difference between left and right brain of the CTZ rats (Figures 5(c) and 5(d)). The result showed that GAT significantly reduced in all the hippocampal subregions, similar to the GAD, in the chronic phase of the CTZ seizure model.

### 3.5. Downregulated BDNF-TrkB Signaling Pathway in Chronic Recurrent Phase in Hippocampus of CTZ Seizure Rats

**Figure 6:**
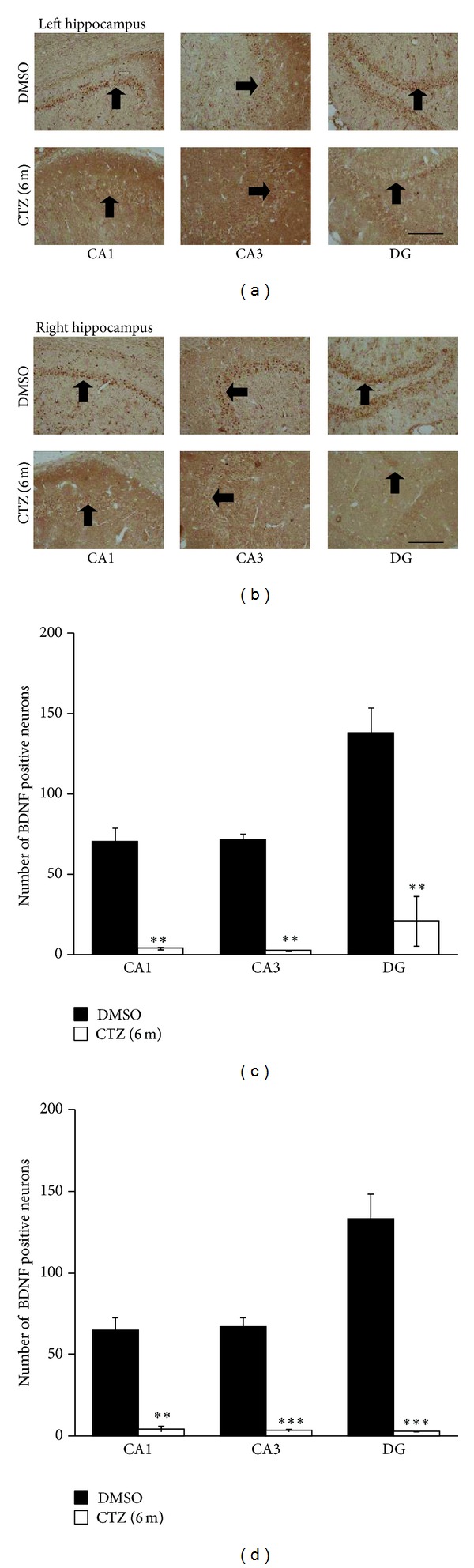
Decreased BDNF staining in hippocampus of recurrent seizure rats 6 months after seizure induction by CTZ. (a-b) Pictures showing BDNF positive cells (arrow indicated) in left (a) and right (b) hippocampus from rats 6 months after either DMSO (top) or CTZ (bottom) treatment. (c-d) Group data showing significant decreases of the BDNF staining cells in CA1, CA3, and DG area of the hippocampus from rats 6 months after either DMSO (*n* = 3) or CTZ (*n* = 3) treatment. **P < 0.01, ***P < 0.001, compared to the DMSO control group. Scale bar in (a) and (b): 200 *μ*m.

We have previously demonstrated that BDNF-TrkB signaling pathway is involved in the induction of the epileptiform activities induced by CTZ in hippocampus [7]. In the current study, we further investigated whether BDNF-TrkB signaling pathway was remaining affected in the chronic recurrent phase in CTZ seizure rats. Immunohistochemistry study showed that the BDNF positive particles existed in the cytoplasm of cells which localized in CA1, CA3, and DG regions close to the pyramidal neuron and granule cells layer, as well as in the molecular and oriens layer of the hippocampus (Figures 6(a) and 6(b)) in DMSO control rats. However, BDNF positive particle containing cells significantly decreased in CA1, CA3, and DG region in CTZ seizure rat hippocampus 6 months after CTZ injection. The number of the BDNF positive neurons was 4.1 ± 1.0 in CA1, 2.9 ± 0.1 in CA3, and 21.2 ± 16.2 in DG in counted area per left hippocampal slice in 3 CTZ rats with recurrent seizure 6 months after CTZ injection, which was significantly reduced in comparison with the time matched DMSO control rats (70.7 ± 9.0 in CA1, 72.2 ± 3,7 in CA3, and 138.3 ± 15.9 in DG (*n* = 3), resp.; *P* < 0.01, *P* < 0.001) (Figure 6(c)). There was also no left right difference in CTZ rats (Figures 6(c) and 6(d)).

**Figure 7:**
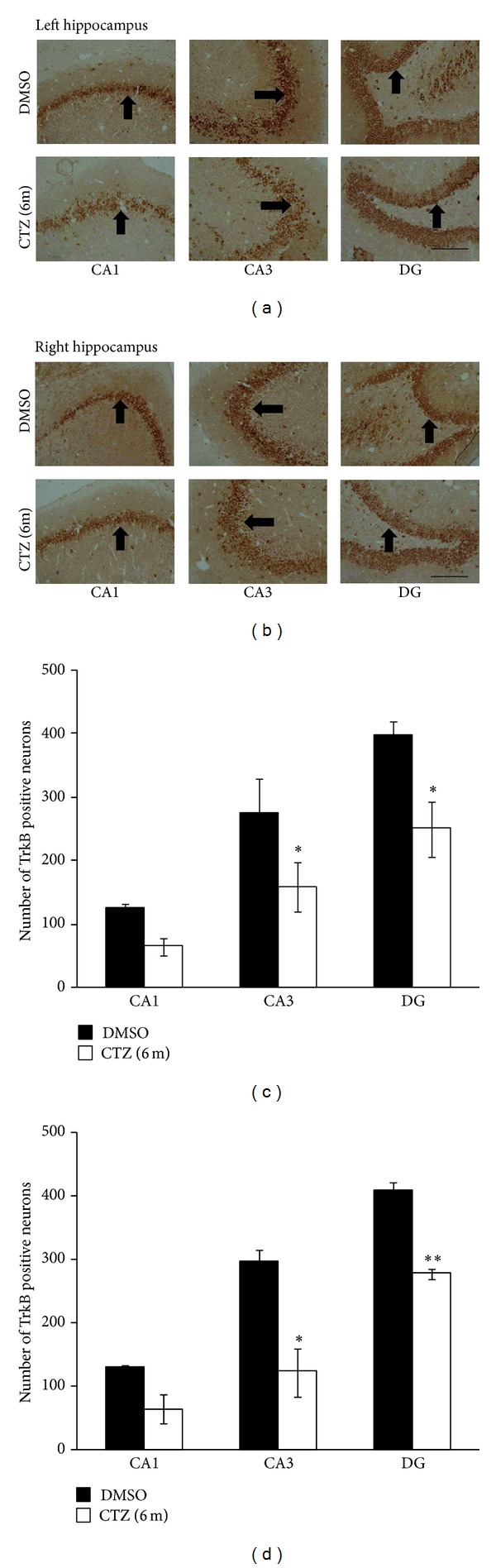
Decreased TrkB staining in hippocampus of recurrent seizure rats induced by CTZ. (a-b) Pictures showing TrkB positive cells (arrow indicated) in left (a) and right (b) hippocampus from rats 6 months after either DMSO (top) or CTZ (bottom) treatment. (c-d) Group data showing significant decreases of the TrkB staining cells in CA1, CA3, and DG area of the hippocampus from rats 6 months after either DMSO (*n* = 3) or CTZ (*n* = 3) treatment. *P < 0.05, **P < 0.01, compared to the DMSO control group. Scale bar in (a) and (b): 200 *μ*m.

In addition, the expression of TrkB in hippocampus of the CTZ rats was also studied as TrkB is known to mediate BDNF effect for epileptogenesis in various seizure models [6, 22, 23], including CTZ induced acute seizure behavior [7]. TrkB positive particles were found to be distributed in the same area as the BDNF in hippocampal subregions of both DMSO and CTZ treated seizure rats (Figures 7(a) and 7(b)). The number of TrkB positive neurons was 64.7 ± 16.2 in CA1, 158.3 ± 41.7 in CA3, and 250.3 ± 46.8 in DG in the counted area per slice of left hippocampus in 3 CTZ rats with recurrent seizure behavior, which was significantly less than those in DMSO control rats (*n* = 3) with the count of 126.7 ± 7.2 in CA1, 275.7 ± 56.3 in CA3, and 399.2 ± 22.4 in DG (*P* < 0.05, *P* < 0.01, except CA1 region) (Figure 7(c)). There was also no left right preference of the TrkB staining change observed in this study (Figures 7(c) and 7(d)).

These data indicate that the BDNF-TrkB signaling pathway possibly has been damaged in long term in the chronic phase of the CTZ seizure models.

## 4. Discussions

In this study, we demonstrated that, in chronic recurrent seizure phase, a large proportion of the rats with acute seizure induced by CTZ exhibited spontaneous seizure behaviour accompanied with epileptic EEG. The immunohistochemistry results, in addition, showed that the number of GABAergic neurons expression of both GAD and GAT was substantially decreased in hippocampus, which would lead to weak GABA inhibitory function in those rats. In addition, a proepileptic signaling pathway, BDNF-TrkB signaling pathway, in acute seizure induction phase [7] was also seen to be downregulated in the chronic phase in comparison with the DMSO control rats.

### 4.1. Characterization of the CTZ Induced Chronic Seizure Animal Model

Previously we have studied the probability of the CTZ as a convulsant with the advantage of its dual action property of simultaneous acting on both glutamate excitatory receptors and GABAA inhibitor receptors [8] to induce epileptiform activities in hippocampal neurons [9, 10, 13]. Although we have demonstrated that CTZ induced seizure in freely moving rats [12], a successful seizure animal model requires the animals to develop recurrent seizure and also have a high proportion to have recurrent seizure [24]. In this aspect we carried out the current study to investigate whether the acutely induced seizure rats by CTZ could develop to have recurrent seizure. Indeed, we found that over 46% of the CTZ rats (6 out of 13) had recurrent seizure with the latency between 2 weeks to 6 months. The silent period between acute seizure induction and the occurrence of the recurrent seizure in this CTZ seizure rat model seems well in agreement with the other classic seizure animal model, at around two-week time of latency, such as pilocarpine and Kainic acid induced seizure models [24, 25]. However, the rate of the animal to have recurrent seizure seems lower than other seizure models. We believe this is due to the underestimating of the rats having recurrent seizure in our current study by using the experimental paradigm of recording 2 hours a day of the seizure behavior during the whole 6-month observation period. Indeed, in 2 rats which were video recorded day and night continuously for 6 months, the recurrent seizure was captured in both of them and also showed multiple recurrent seizure episodes (3 and 4 episodes, resp.) in the 6-month period. In this aspect, we cannot fully rule out that the 4 animals without recurrent seizure behavior observed were “nonchronic seizure” rats. In addition, among 4 out of the 13 rats that died between 15 and 99 days after initial CTZ seizure induction, one of the rats, by chance, was observed to have generalised seizure (Racine score V behavior) and died at the next day. In this aspect, we hypothesise that the other 3 dead rats possibly had also seizure episode(s) which was the cause for their death, since none of the DMSO control rats (*n* = 8) died during our 6-month experimental period. Taking these together, we believe that the CTZ induced seizure rats would have much higher recurrent seizure occurrence rate. However, this needs to be studied in the future in more careful design of the experiment protocol.

### 4.2. Decreased GABAergic Transmission in Chronic Phase of the CTZ Seizure Rats

CTZ not only blocks AMPA receptor desensitization, but also inhibits GABAA receptors [8], and GABAA receptors were involved in the CTZ induced epileptiform activities in hippocampal neurons [9–11, 13]. In this study, we reported further that, at the chronic phase of the CTZ seizure rat, there was a significant reduction of the GAD and GAT containing neurons in the hippocampus. Lack of GAD may lead to the weakened GABA inhibition and increased probability of the seizure occurrence. Indeed, our preliminary result on determination of the amount of GABA in the hippocampus in the chronic CTZ seizure rats with GABA antibody showed that the optical density for GABA staining was lower in neurons of CA1, CA3, and DG of CTZ seizure models than those of DMSO rats (data not supplied). Decreased GABA may cause the imbalance of excitation and inhibition, which could be one of the reasons to cause the recurrent spontaneous seizure.

GABA is the primary inhibitory neurotransmitter in the adult brain, and its effects at receptors are primarily terminated by diffusion and transportation by GABA transporters, GAT-1 and GAT-3. They are primarily localized to presynaptic terminals and to glial processes adjacent to the synaptic cleft [18–20]. There are controversial opinions about expression and mechanism of GAT-1 in hippocampus after seizure. Although the exact mechanism for a decrease in transporter is unknown, it is interesting to note that the expression of this protein is dependent on the extracellular GABA level [26]. In this study, we observed that GAT-1 became reduced in hippocampus in chronic phase of the CTZ rats, which had a similar pattern as the loss of the GABA synthesis protein (GAD) reduction in the hippocampus in chronic CTZ rats. Thus, reduced GAT-1 may serve as a compensatory mechanism for reduced GABA release, which would partly reverse the GABA inhibitory function in the recurrent spontaneous seizure phase.

### 4.3. Downregulated BDNF-TrkB Signaling Pathway Induced Recurrent Spontaneous Seizure in CTZ Model Rats

Epileptogenic insults increase BDNF synthesis and TrkB receptor activation [5, 27]. Many studies convincingly support the notion that these phenomena have a proepileptogenic role [6, 27]. The previous in vitro and in vivo study also showed that, during CTZ induced acute seizure, BDNF-TrkB signaling pathway is activated to promote the epileptiform activities in hippocampal neurons [7]. However, our current results demonstrated that BDNF decreased significantly in CA1, CA3, and DG and its receptor, TrkB, also decreased with less extent in chronic seizure phase in CTZ rats. It has recently been demonstrated that epileptogenic seizures promote accumulation of selected BDNF splice variants into the distal dendritic compartment whereas others remain restricted to the cell soma [28, 29]. BDNF in soma may favor survival or regeneration (or both) of hippocampal neurons damaged by seizure [30]. BDNF also can amplify GABA currents in oocytes expressing GABAA receptors transplanted from surgically removed specimens of human epileptic brains [31]. In this study, decreased BDNF occurred in soma of neurons in hippocampus, and neurotrophic action was impaired and could not amplify GABA currents. Thus, the downregulated BDNF-TrkB signaling pathway may suggest involving in inducing recurrent spontaneous seizure in CTZ model rats.

## 5. Conclusion

In the current study, we investigated in detail the occurrence of the recurrent seizure behavior for up to 6 months in CTZ induced seizure rats. Results demonstrated that high proportion of the acute seizure animals induced by CTZ could develop to chronic seizure, which was associated with the damaged GABA inhibitory system and BDNF-TrkB signaling pathway. This study provides further evidence for using CTZ as a convulsant for epilepsy study and CTZ rats may serve as an animal model for anticonvulsant drug test.

## Figures and Tables

**Table 1:** Recurrent seizure behavior (Racine score IV-V behavior) occurrence in the chronic phase in CTZ induced seizure rats.

Group	*n*	Mortality rate	Racine III recurrent seizure rate	Racine IV-V recurrent seizure rate	Seizure IV-V latency (days)	Acute seizure score	Recurrent seizure score
CTZ	13	30.8% (*n* = 4/13)	46.2% (*n* = 6/13)	46.2% (*n* = 6/13)	76.3 ± 24.8 (*n* = 6)	4.75 ± 0.16*** (*n* = 10)	2.60 ± 0.72* (*n* = 10)
DMSO	8	0% (*n* = 0/8)	12.5% (*n* = 1/8)	0% (*n* = 0/8)	—	0 (*n* = 8)	0.38 ± 0.38 (*n* = 8)

*P < 0.05, ***P < 0.001, in comparison with the DMSO control.
